# Scopolamine for Tremor

**Published:** 1909-06-19

**Authors:** 


### SCOPOLAMINE FOR TREMOR.

Scopolamine has been found to be of considerable use in decreasing the tremors of paralysis agitans, and if the complaint is in an early stage the troublesome shakings of the hands may not only be decreased but in some instances actually abolished for the time being. Even in those more decided cases in which this drug only succeeds in lessening the tremor, the latter remains lessened for some days afterwards. The drug is given hypodermically in precisely the same way as one administers a morphia injection; the salt used is the hydrobromide, and the dose may vary from $\frac{1}{250}$ grain to $\frac{1}{100}$ grain once daily.

Some other types of tremor besides that of paralysis agitans may be mitigated by scopolamine hydrobromide, though certain types are scarcely benefited at all. The tremors of general paralysis of the insane, for instance, are not relieved, nor are those of Graves' disease (exophthalmic goître). The movements or shakiness of hereditary tremors, on the other hand, and also those of alcoholic tremor and of disseminated spinal sclerosis, have been much benefited sometimes; so that, although one must not be disappointed if a given case of tremor is not relieved by this treatment, it is a therapeutic measure that is well worth bearing in mind in cases where tremor is a troublesome symptom.

Some patients react to smaller doses of the drug than do others. The susceptibility to it should be ascertained in each case by starting with smaller doses, which may be gradually increased.

